# Supplementary material for: Ficus septica plant extracts for treating Dengue virus in vitro
Source: PeerJ. 2017 Jun 8;5:e3448. doi: 10.7717/peerj.3448 (PMC5466810; doi:10.7717/peerj.3448)
Supplement: Table S1 [file peerj-05-3448-s001.docx]

**Supplementary information**

Table S1. The cytotoxicity of plant crude extracts in A549 cells

| **No.** | **Botanical name** | **Part of plant** | **Extract** | **Abbreviation of crude extract** | **IC_50_ (μg/ml) of cell growth inhibition** | **Extract excluded in this study** |
| --- | --- | --- | --- | --- | --- | --- |
| 1 | *Alisma orientalis* | Whole plant | Methanol | Ao-(WP)-M | > 100 |  |
| 2 | *Asparagus cochinchinensis* (Lour.) Merr. | Root | Methanol | ACM (R)-M | > 100 |  |
| 3 | *Broussonetia papyrifera* | Leaves | Methanol | BP (L)-M | > 100 |  |
| 4 | *Catharanthus roseus* | Whole plant | Methanol | CaR-(WP)-M | > 100 |  |
| 5 | *Clausena excavata* | Leaves | Methanol | Ce-(L)-M | > 100 |  |
| 6 | *Cinnamomum insulari-montanum* | Leaves | Methanol | CiM-(L)-M | > 100 |  |
| 7 | *Cornus officinalis* | Whole plant | Acetone | CO -(WP)-A | > 100 |  |
|  |  | Whole plant | Methanol | CO-(WP)-M | > 100 |  |
| 8 | *Euonymus japonicus* | Leaves | Acetone | EJa-L-A | > 100 |  |
|  |  |  |  |  |  |  |
| 9 | *Elaeocarpus sylvestris* | Leaves | Acetone | ES-(L)-A | > 100 |  |
|  |  | Leaves  Leaves | Chloroform  Methanol | ES-(L)-C  ES-(L)-M | > 100  > 100 |  |
| 10 | *Fraxinus griffithii* | Leaves  Leaves  Leaves | Acetone  Chloroform  Methanol | FG-(L)-A  FG-(L)-C  FG-(L)-M | > 100  > 100  > 100 |  |
| 11 | *Ficus septica* | Root Bark | Acetone | FS-(RB)-A | > 100 |  |
|  |  | Leaves | Methanol-Ethyl acetate | FS-(L)-M-ET | > 100 |  |
|  |  | Fruit | Methanol | FS-(F)-M | > 100 |  |
|  |  | Heartwood  Leaves  Leaves  Leaves  Stem | Methanol  Acetone  Chloroform  Methanol  Methanol | FS-(HW)-M  FS-(L)-A  FS-(L)-C  FS-(L)-M  FS-(S)-M | > 100  > 100  > 100  > 100  > 100 |  |
| 12 | *Ficus sarmentosa* var. *henryi* | Leaves | Acetone | FSVH-(L)-A | > 100 |  |
|  |  |  |  |  |  |  |
| 13  14 | *Garcinia subelliptica*  *Lumnitzera racemosa* | Flower | Methanol | GS-(F)-M | > 100 |  |
|  |  | Leaves | Methanol | Lr-(L)-M | > 100 |  |
| 15 | *Litchi chinensis* | Leaves | Acetone | LC-(L)-A | > 100 |  |
|  |  | Leaves | Chloroform | LC-(L)-C | > 100 |  |
|  |  | Leaves | Methanol | LC-(L)-M | > 100 |  |
|  |  | Stem  Stem  Stem  Fruit  Pericarp  Pericarp | Acetone  Chloroform  Methanol Acetone  Acetone  Methanol | LC-(S)-A  LC-(S)-C  LC-(S)-M LC-(FR)-A  LC-(Peri)-A  LC-(Peri)-M | > 100  > 100  > 100  > 100  > 100  > 100 |  |
| 16 | *Phytolacca americana* | Whole plant  Whole plant | Acetone  Chloroform | PA-(WP)-A PA-(WP)-C | > 100  > 100 |  |
| 17 | *Pueraria lobata* | Whole plant | Methanol | PL -(WP)-M | > 100 |  |
| 18 | *Sida acuta* | Whole plant | Methanol | Sa-(WP)-M | > 100 |  |
| 19 | *Sambucus chinensis*Lindl | Whole plant | Chloroform | Scl-(WP)-A | > 100 |  |
|  |  | Whole plant | Methano | Scl-(WP)-C | > 100 |  |
|  |  | Whole plant | Methanol | Scl-(WP)-M | > 100 |  |
| 20 | *Scrophularia ningpoensis* | Whole plant | Methanol | SN-(WP)-M | > 100 |  |
|  |  |  |  |  |  |  |
| 21 | *Saurauia tristyla* var. *oldhamii* | Leaves | Methanol | STV-(L)-M | > 100 |  |
| 22 | *Tribulus terrestris* | Fruit  Fruit  Fruit  Whole plant  Whole plant  Whole plant | Acetone  Methanol  Chloroform  Acetone  Chloroform  Methanol | TT-(Fr)-A  TT-(Fr)-M  TT-(Fr)-C  TT-(WP)-A  TT-(WP)-C  TT-(WP)-M | > 100  > 100  > 100  > 100  > 100  > 100 |  |
| 23 | *Xanthium sibiricum* | Fruit  Fruit | Chloroform  Methanol | XS-(Fr)-C  XS-(Fr)-M | > 100  > 100 |  |
| 24 | *Strophanthus divaricatus* | Stem xylem  Stem xylem  Stem bark  Stem bark | Acetone  Chloroform  Acetone  Methanol | SD-sx-A  SD-sx-C  SD-sb-A  SD-sb-M | **< 3.125**  **< 3.125**  **< 3.125**  **< .125** | **excluded excluded excluded excluded** |
